# Multiple micronutrient supplements in pregnancy: Implementation considerations for integration as part of quality services in routine antenatal care. Objectives, results, and conclusions of the meeting

**DOI:** 10.1111/mcn.12704

**Published:** 2018-12-26

**Authors:** Maria Nieves Garcia‐Casal, Diana Estevez, Luz Maria De‐Regil

**Affiliations:** ^1^ Evidence and Programme Guidance Unit, Department of Nutrition for Health and Development World Health Organization Geneva Switzerland; ^2^ Nutrition International, Division of Global Technical Services Ottawa Ontario Canada

**Keywords:** folic acid, iron, meeting, multiple micronutrient supplements, pregnancy, public health programmes

## Abstract

Health promotion, screening, diagnosis, and disease prevention are essential services of quality routine antenatal care for pregnant adult and adolescent women. Supplementation programmes in pregnancy, generally implemented in the context of antenatal care services, have had less than optimal results in many countries, generally attributed to limited access, low coverage, and reduced adherence to the recommended regimens and counselling. The World Health Organization Department of Nutrition for Health and Development, in collaboration with the United Nations Children's Fund and Nutrition International, convened the technical consultation “Multiple micronutrient supplements in pregnancy: Implementation considerations for successful incorporation into existing programmes.” The objectives of the technical consultation were to (a) examine implementation experiences of micronutrient supplementation interventions in pregnant women, lessons learnt, and best practices; (b) discuss programmatic and technical considerations of interventions on multiple micronutrient supplementation in pregnant women in low‐, middle‐, and high‐income countries; and (c) identify implementation considerations that can be useful to scaling up efforts by national policymaker and their advisors considering multiple micronutrient supplementation in pregnant women as part of existing antenatal care programmes as well as other delivery platforms. The consultation was based on presentations of background papers, case studies, and plenary discussions. Country representatives were asked to discuss the context of micronutrient supplementation for their countries and share implementation challenges they faced. This paper provides the background and rationale of the technical consultation, synopsises the presentations, and provides a summary of the main considerations and conclusions reached during plenary discussions.

**Key messages**

- Micronutrient malnutrition persists among childbearing aged and pregnant women worldwide.
- Successful antenatal supplementation programmes require addressing policy, operational, and financial issues in addition to selecting an appropriate nutrition commodity, which should respond to the nutrition and health burden in pregnancy and post birth.
- Micronutrient supplementation during pregnancy can benefit from setting up or strengthening monitoring and surveillance systems.
- Creating awareness and knowledge about optimal nutrition before and during pregnancy augments the potential to beneficially impact pregnancy outcomes.

## 1 INTRODUCTION

Globally, micronutrient malnutrition in pregnant women is widespread across regions and countries. It is estimated that approximately 32 million pregnant women are anaemic worldwide and 19 million suffer from vitamin A deficiency (Ahmed, Hossain, & Sanin, 2012; Muthayya et al., 2013; World Health Organization, 2015, 2016a). Vitamin and mineral deficiencies have been associated with pregnancy complications and poor birth and infant outcomes (Gernand, Schulze, Stewart, West, & Christian, 2016). It is calculated that approximately 20 million babies are born weighing less than 2,500 g at birth (low birth weight [LBW]), about 15 million are premature, and many more are born small for their gestational age, increasing their risk of morbidity and mortality during childhood (Seshadri, 2001; World Health Organization, 2014).

Along with other nutrition and health interventions at population level, the World Health Organization (WHO) has recommended supplementing pregnant women with iron and folic acid (IFA) to prevent and treat gestational anaemia since 1968. Currently, two WHO guidelines related to pregnancy and postpartum (World Health Organization, 2016b, 2016c) advise that both interventions be part of the quality services of routine antenatal and postnatal care. Though the use of multiple micronutrient (MMN) supplementation is not recommended routinely for all pregnant women to improve maternal and perinatal outcomes, the WHO has indicated that policymakers in populations with a high prevalence of nutritional deficiencies might consider the benefits of MMN supplements on maternal health in comparison with IFA alone.

In spite of the evidence supporting the efficacy of these interventions, supplementation programmes in pregnancy, generally implemented in the context of antenatal care (ANC) programmes, have had less than optimal results in many countries, including low intervention coverage and adherence. Reasons, among others, include women's limited access to routine and timely ANC due to geographic distance, reduced number of facilities and other gender‐related factors affecting women's access to healthcare, including their beliefs and motivation about the daily use of the supplements and their own expectations of care (Bhutta et al., 2013; Risnes et al., 2011). In other settings, low motivation, poor interpersonal skills, and training of health staff have been limiting factors. Poor quality and insufficient supply of supplements, due to inadequate programme contextualization, may also affect programmes' success (Abu‐Saad & Fraser, 2010; March of Dimes, 2012).

Owing to the increased needs of various vitamins and minerals during pregnancy, WHO remarks that IFA formulations may also include other vitamins and minerals in a MMN supplement. This intervention is being implemented in a few low‐, middle‐, and high‐income countries and has also been the practice in emergency settings. There is evidence suggesting that there may be decreased risk of LBW and small‐for‐gestational age in comparison with IFA supplementation alone (Haider & Bhutta, 2017; Ramakrishnan et al., 2012). However, before considering the implementation of this nutrition intervention as part of routine ANC at large scale, particularly in low‐ and middle‐income countries, it is necessary to examine key implementation considerations for helping interested parties to successfully incorporate this intervention into existing programmes.

## 2 TECHNICAL CONSULTATION

The Evidence and Programme Guidance Unit, Department of Nutrition for Health and Development, WHO, in collaboration with the United Nations Children's Fund (UNICEF) and Nutrition International, convened the technical consultation “Multiple micronutrient supplements in pregnancy: Implementation considerations for successful incorporation into existing programmes,” to examine the programmatic evidence, including successful implementation experiences, best practices, and lessons learnt, in order to inform the scale up of MMN supplementation during pregnancy as part of quality existing services in routine ANC programmes in these settings. The objectives of the technical consultation were to (a) examine implementation experiences of micronutrient supplementation interventions in pregnant women, lessons learnt, and best practices; (b) discuss programmatic and technical considerations of interventions on MMN supplementation in pregnant women in high‐, middle‐, and low‐income countries; and (c) identify the needed implementation considerations that can be useful to scaling up efforts by member states considering MMN supplementation in pregnant women as part of existing ANC programmes as well as other delivery platforms.

Prior to this technical consultation, the Evidence and Programme Guidance Unit, launched a call for authors interested in preparing review papers on diverse topics related to the implementation considerations for the successful integration of MMN supplements in pregnancy into existing programmes. The papers and case studies reviewed technical areas and evidence gaps to provide policy makers with guidance on micronutrient supplementation interventions in pregnant women. Authors were requested to gather programmatic evidence including successful implementation experiences, best practices, and lessons learnt that could provide policy makers with the best available evidence to inform policies and programmes providing MMN supplements to pregnant women.

The topics requested in the call for authors included an overview of the major policy‐related considerations related to IFA and MMN supplementation, including delivery platforms; the identification of the major cost‐related concerns involved in introducing MMN and in transitioning from IFA to MMN; the production and procurement processes likely to be affected by a transition from IFA to MMN; the implications for demand creation identifying the mechanisms needed to introduce MMN or shift from IFA to MMN on a programmatic/operational level when countries decide to introduce MMN; surveillance and monitoring systems detailing programme indicators needed to be collected on a regular basis to track progress on the introduction of MMN or on the transition from IFA to MMN and their implementation; the ethical implications and health equity concerns in implementing MMN; programmatic experiences of MMN interventions in high‐, middle‐, and low‐income countries; and programmatic experiences on IFA supplementation that could provide useful learnings for the introduction of MMN.

The consultation was based on, but not limited to, the background papers and case studies that were commissioned through the public call for papers. It included the presentation of the commissioned papers and other topics of interest and plenary discussions. Country representatives were asked to prepare 5‐min presentations discussing the context of micronutrient supplementation for each country and what implementation challenges they faced.

## 3 PRESENTATIONS AND DISCUSSIONS

### 3.1 Global estimates of micronutrient malnutrition in pregnancy

Monitoring the health situation and assessing global health trends are two of WHO core functions. Regarding vitamin and mineral status, in 1991, the WHO Department of Nutrition developed a Micronutrients Database as part of its Vitamin and Mineral Nutrition Information System, which was previously called the Micronutrient Deficiency Information System. This database compiles national, subnational, and first administrative level data on the vitamin and mineral status of populations that is used to monitor the micronutrient status of populations, to provide global estimates of the burden of vitamin and mineral deficiencies, and to calculate trends in status over time. WHO, along with partner agencies periodically uses the data from the Micronutrients Database to develop global estimates of vitamin and mineral deficiencies to fill a gap for countries that do not have data, to identify high‐priority areas to target and implement micronutrient interventions, to advocate for resource allocation, and to assess the influence of vitamin and mineral deficiencies as risk factors to the overall global burden of disease.

The global estimates of anaemia and vitamin A deficiency in pregnancy were presented. The estimated anaemia prevalence in pregnant women changed from 43% in 1995 to 38% in 2011, with 32 million pregnant women affected globally (Stevens et al., 2013). For vitamin A, the available global estimates for pregnant women were from 2009 (World Health Organization, 2009) and assumed that vitamin A deficiency was not a public health problem in 37 countries with a GDP ≥ 15,000 USD, although about 8% (9.8 million) of the world's population of pregnant women were estimated to be night blind and 15% (19.1 million) had serum retinol <0.70 μmol/L. The Africa and Southeast Asia regions had the highest risk of deficiency and carried the majority of the burden (World Health Organization, 2009). The audience pointed out the relevance of measuring and reporting on additional micronutrient deficiencies among pregnant women, including iodine deficiency, for which there are estimates in other age groups.

### 3.2 WHO ANC guidelines

At the moment of this technical consultation, WHO recommendations on ANC for a positive pregnancy experience were in the process of development. The document was published in 2016 (World Health Organization, 2016b) and contains 39 recommendations related to five types of interventions: (a) nutritional interventions, (b) maternal and fetal assessment, (c) preventive measures, (d) interventions for common physiological symptoms, and (e) health systems interventions to improve utilization and quality of ANC. In addition, ANC relevant recommendations from current guidance produced by other WHO departments were systematically identified, and 10 such recommendations were consolidated into the guideline (to a total of 49 recommendations) for the purpose of providing a comprehensive document for pregnant women and adolescent girls. The guideline responded to issues surrounding the practice and delivery of ANC and to prioritised person‐centred health and well‐being.

### 3.3 An evidence update on the effects of MMNs in pregnancy in comparison with iron folate supplements

The systematic review evaluated the benefits of oral MMNs (MMN including IFA) provision during pregnancy on maternal, fetal, and infant outcomes in comparison with current standard of care (Haider & Bhutta, 2017). Data from 23 trials (involving 139,560 pregnant women at varying gestational stages) were included, 16 trials providing IFA, and four supplementation trials assessed MMN against placebo and IFA. All supplements were given orally to the pregnant women throughout pregnancy from the time of enrolment.

Authors' findings suggested that MMN with iron and/or folic acid were effective in reducing maternal anaemia and improving birth outcomes and that MMN had comparable effects on anaemia reduction than IFA used alone but greater benefits in reducing the risk of LBW and small for gestational age, with a small effect on reducing stillbirths. Also, authors suggested the replacement of IFA in pregnant women in countries where MMN deficiencies are common, with MMN containing IFA and that this intervention should be integrated in maternal nutrition and ANC programmes in developing countries and in fostering studies of effectiveness and cost‐effectiveness of this intervention in programmatic settings.

### 3.4 Progress on the comprehensive implementation plan on maternal infant and young child nutrition

A review and policy analysis of member states in 2009–2010 indicated that most countries have a range of policies and programmes on nutrition. However, such policies are often inadequate in face of the complexity of the challenges of maternal, infant, and young child nutrition and do not produce the expected impact. Many countries have adopted integrated strategies for maternal, newborn, and child health that incorporate nutrition interventions, but the actual delivery of nutrition support in health services is often inadequate, and few indicators are available to measure the coverage (World Health Organization, 2014).

The comprehensive implementation plan on maternal, infant, and young child nutrition aims to alleviate the double burden of malnutrition in children, starting from the earliest stages of development (World Health Organization, 2012, 2014). Substantial benefits can be obtained by concentrating efforts from conception through the first 2 years of life, but at the same time, a life‐course approach needs to be considered so that good nutritional status can be maintained. Progress can be made in the short term, and most nutrition challenges can be resolved within the current generation (Bhutta et al., 2008). The best way is to set targets at country level, assess the resources available, ensure development policies and programmes include nutrition, create links between different sectors as well as different stakeholders, and develop and implement suitable monitoring and evaluation mechanisms.

Evidence‐based, effective interventions, both nutrition specific and nutrition sensitive investments at the policy, health system, and community levels are needed. Some strategies should include identifying, scale‐up coverage of prevention and treatment, developing strategic interventions, monitoring, and evaluating. Beyond these, there are other collaborative approaches, such as the 10 commitments of the FAO/WHO Second International Conference on Nutrition (ICN2), Every Woman Every Child, 1,000 days, Framework for Action, and the commitment of the member states in the last World Health Assembly.

### 3.5 Overview of major policy‐related considerations on IFA and MMN supplementation, including delivery platforms

The objective was to review existing policies (action plans, strategies, or guidelines) on IFA supplementation, MMN supplementation, and supplementation with other micronutrients. The policy search was performed at national level, and the review was limited to those countries with the highest burden of LBW. Twenty countries with the highest prevalence of LBW, according to Demographic Health Surveys and Multiple Indicator Cluster Survey data, and a minimum population of 1 million were included in analysis. Thirty‐eight policies were reviewed. Four countries, Ethiopia, Nigeria, Mozambique, and Madagascar, included MMN supplementation in their policies. All policies referred to universal iron supplementation during pregnancy. There were also some policies for adolescent girls and women in reproductive age. Twelve countries referred that supplementation helped prevent LBW (Pakistan, Yemen, India, Haiti, Bangladesh, Ethiopia, Mali, Nepal, Sri Lanka, Mozambique, Namibia, Nigeria).

Conclusions from this review indicated that 19 out of 20 countries had policies for IFA and that although four countries considered MMN an alternative to IFA, there was limited information on dose or supplement composition. The results also showed that ANC was the main delivery platform, and although community‐based approaches existed, they were poorly implemented. The information on other delivery platforms, for example, child health checks or malaria prevention programmes, was limited.

### 3.6 Micronutrient supplementation among pregnant women: A review of the evidence from large‐scale prenatal micronutrient programmes on factors contributing to success or failure in coverage, compliance, and impact

This commissioned review aimed at gathering and review available experiences from large‐scale prenatal micronutrient supplementation interventions in order to highlight factors, determinants, and conditions contributing to success or failure of interventions. Authors extracted information on implementation, coverage, compliance ,and impact from reports of large scale interventions in Central America, Southeast Asia, South Asia, and Sub‐Saharan Africa. Findings suggested that to successfully implement supplementation interventions and achieve sustainable, permanent solutions efforts must focus on factors and processes related to quality, cost‐effectiveness, coverage, utilisation, demand, outcomes, impacts, and sustainability of programmes including strategic analysis, management, collaborations to pilot a project, careful monitoring, mid‐course corrections, supervision, and logistical support to gradually scaling it up (Berti, Gaffey, Bhutta, & Cetin, 2018).

### 3.7 Planning tools for implementing evidence‐informed nutrition policies and programmes: GINA and the OneHealth Tool

The global database on the implementation of nutrition actions (GINA) is a database that contains more than 3,500 national nutrition‐related policies and actions. It provides a repository of policy commitments made in relation to nutrition and lessons learnt from country implementations (http://www.who.int/nutrition/gina/en/).

United Nations (UN) OneHealth Tool was developed by the UN Inter‐agency Working Group on Costing, sector wide strategic health planning, infrastructure, logistics, scenario analysis, and is linked to health impact analysis (Life Save Tools) to plan, cost, and prioritise nutrition programmes (http://www.avenirhealth.org/software-onehealth.php). For example, it includes MMNs supplementation as one alternative option for implementation compared with daily IFA supplementation in pregnant women: IFA costs per average‐case = 0.91 USD/MMN costs per average‐case = 2.98 USD.

### 3.8 Health equity concerns on the implementation of MMN supplementation during pregnancy

This presentation provided an overview of considerations between research, policy, and programmes from the equity perspective. Health inequities interact with each other, and they reinforce each other, such is the case of severe micronutrient deficiencies.

Research on health inequities is scarce, and systematic reviews do not report on health inequities very often, and although there are tools and methods to fill this gap, evidence‐informed policy making will benefit from more research on inequities and from systematic reviews reporting the effects of inequities in outcomes. There are some proposed tools and methods to report health inequities in systematic reviews, for example, PROGRESS‐Plus (O'Neill et al., 2014).

Programmatic implementation must consider social determinants of health‐ and equity‐oriented implementation research can better inform scaling up policies and actions.

### 3.9 IFA supplementation in pregnant women: Barriers, enablers from multi‐country formative assessments and implications for future supplementation programmes

This review aimed to identify barriers and potential‐enabling opportunities for improved coverage and adherence by pregnant women who take IFA supplementation during pregnancy and to reflect on implications for large scale supplementation programmes, including MMN (Siekmans, Roche, Kung'u, Desrochers, & De‐Regil, 2018).

The formative research process followed the identification of enablers and barriers, implementation and delivery, coverage and adherence, and outcomes during pregnancy. The search was conducted in selected districts/sites of Afghanistan, Bangladesh, Ethiopia, Indonesia, Kenya, Nigeria, and Senegal between 2012 and 2013 to better understand IFA supplementation knowledge, attitudes, and practices among pregnant women and women with young children, their family members, and health care providers.

Barriers to timely access to IFA supplements were closely related to those for seeking ANC in the first trimester. Few women knew the importance of or saw value in seeking ANC during the first trimester. Women in Ethiopia and Senegal expressed concern with revealing the pregnancy so early, because this could put the fetus at risk (Siekmans et al., 2018). Relational barriers to early ANC attendance were also related to social implications of revealing a pregnancy and the support of their husband and/or mother‐in‐law. Authors concluded that improved ANC access and quality is needed to facilitate MMN supplementation. Community‐based delivery and counselling; behaviour change interventions to target women all ages, men, and health care providers; renewed investment; and efforts in prenatal supplementation are needed (Siekmans et al., 2018).

### 3.10 SORT IT: The potential of operational research for nutrition programmes

SORT IT is a tool for operational research with advocacy power, which aims to help countries to conduct operational research in accordance with their own priorities, to develop adequate and sustainable operational research capacity in public health programmes, and to create an organizational culture of policy and practice that is informed by operational research and leads to improved programme performance.

The process involves the discussion with the countries who need training and capacity building, then develops operational research protocol around the priorities identified, workshops and mentorship to build data files, analysis, and new workshop to write a paper for publication. With these results, communication strategies and returning to the countries to be transformed in policies ensuring that practical issues of this research are implemented or considered. More information about this tool at http://www.who.int/tdr/capacity/strengthening/sort/en/

### 3.11 Approaches to analysing barriers to effective coverage and health equity‐potential relevance for micronutrient supplementation in pregnancy

Because the barriers to effective coverage and health equity are influenced by the health sector, other sectors, and the communities (Hirmas Adauy et al., 2013; Siekmans et al., 2018), this presentation detailed the Tanahashi framework for effective coverage that goes from establishing the service delivery goal to reach the target population and requires the assessment of a coverage curve that includes effectiveness, contact, acceptability, accessibility, and availability of coverage (Tanahashi, 1978).

### 3.12 IFA supplementation during pregnancy: Sri Lankan experience

In Sri Lanka (population 20.3 million), health services are provided totally free for end users: from minor ailment to complicated surgery with all drugs and investigations. Education is free for all from grade 1 to under graduate degree. The accessibility to services is good, with a complete road network (Department of Census and Statistics, 2017).

Maternal and child health care system is a special platform that takes care of the cycle of life, guided by the family and health bureau. The couple have three compulsory ANC visits (one for each trimester). Coverage of pregnant women receiving ANC is almost 100%, and the average number of ANC clinic visits is between 12 and 15. Ninety‐six percent of deliveries occur in government hospitals and 4% in private clinics. IFA supplementation for pregnant women is usually done during antenatal clinic care, both in public and private facilities. More than 95% of pregnant women receive their first antenatal clinic visit before 12 weeks of gestation. Up to 12 weeks of gestation, they are provided only with folic acid supplementation. After that, both iron and calcium supplementation together with vitamin C are provided.

Currently, the programme uses ferrous sulphate (200 mg), which is imported through global open tender procedure, and folic acid tablets (1 mg), which are locally manufactured. The combined pill was yet to come at the moment of this meeting. The national policy is to continue IFA for 6 months after delivery. Monitoring, evaluation, and flexibility have evolved to clear revised guidelines, programmes, and national strategic plans on maternal care and newborn health and the development of national policies (Ministry of Health, 2012). The 2015 survey showed 95.7% of pregnant mothers had received IFA supplements at antenatal clinics. However, only 21% of pregnant mothers have taken 90 or more iron tablets throughout their pregnancy (Medical Research Institute, 2016). The same survey reported a reduction in anaemia prevalence in pregnant women 15 to 49 years of age from 34% in 2006 to 16.7% in 2009 (Medical Research Institute, 2016).

### 3.13 A market‐based approach to MMN interventions in Myanmar and Somaliland

Population Services International social marketing approach consisted in identifying health care market sectors; consumer segmentation; conduct formative research; and market strategy of products, pricing, placement, and promotion. At the moment of the technical consultation, they were finalizing training materials and launching social marketing campaigns and product procurement (Population Services International, 2015a, 2015b).

The presentation was a description of the programme characteristics and the ongoing development within the national policy in Somaliland and Myanmar contexts, together with the social marketing campaign, which included consumer profile generated from consumer insight and brand positioning statements of how MMN will be described and intended to be perceived by consumers.

### 3.14 Situation analysis of production and procurement of MMN supplements in 12 low‐, middle‐, and high‐income countries

This paper presented a situation analysis of the market, manufacturing, and policy factors that were driving the production of MMN in 12 low‐, middle‐, and high‐income countries. Key informants completed a self‐administered structured questionnaire, which examined the local context of products available in the market and their cost, regulations, and policies, in Brazil, Colombia, Guatemala, Mexico, Peru, Bangladesh, India, Vietnam, Ghana, Kenya, Nigeria, and South Africa (Monterrosa et al., 2018). The study found that although most countries have the capacity to produce locally MMS, the major barriers observed for sustainable and affordable production included poor technical capacity and policies for ensuring quality along the value chain and lack of policy coherence to incentivise local production and lower the manufacture and retail price of MMS.

### 3.15 Cost‐effective typologies for successful implementation strategies for front line health workers and demand creation for maternal MMN supplementation

Delivery channels mapping for 27 countries chosen using a specific clustering model considering variables such as health systems, poverty, malnutrition, primary exports, and productivity (Tezanos Vázquez & Sumner, 2013). Regarding IFA and MMN, most of them had these products in the market as sachet presentations, promotion through health workers and printed media, delivery through government services distributed by health workers, and community‐based workers distributed at the health facilities.

Cost in USD per pregnancy was assessed under each delivery channel. MMN is more expensive than IFA. Blister pack is the most expensive presentation, followed by bottle and the least expensive presentation is the sachets (3 USD for IFA and 5 USD for MMN).

The highest cost is attributed to personal distributing to women, followed by the location of distribution (only in this issue, the cost of IFA and MMS are even), and promotion and distribution activities are lower. Average total costs estimate IFA 5.7 USD versus MMN 9.4 USD (mostly manufacturing).

The authors conclude that countries are heterogeneous for delivery channels for IFA and MMN, being promotion activities, the main source of heterogeneity. Predominant costs are generated from the supplements and staff. Across 54 low‐ and middle‐income countries, the transition to MMS yields a 23:1 return on investment at 20% coverage rates, which indicates that transition to MMS could be cost‐effective.

### 3.16 Monitoring and surveillance for MMN supplements in pregnancy

The objective of this review was to describe monitoring, evaluation, and surveillance indicators for MMN in pregnancy programmes by developing indicator titles based on the WHO/CDC logic model to cover the areas of inputs, activities, outputs, and outcomes (World Health Organization/Centers for Disease Control and Prevention, 2016). For this purpose, practice‐based evidence was assessed including monitoring manuals, implementation manuals, country reports, published literature, and expert opinion. Twenty‐eight potential indicators were proposed to cover MMN supplementation programmes, which could be selected and adapted to any setting depending on resources, stakeholders, needs, and priorities. Some key indicators for MMN programmes monitoring included key stakeholders support to MMN programme in pregnancy; existence of a work plan for forthcoming year that addresses supplies, human resources, and budget; sufficient MMN supply at distribution sites; data on coverage of MMN supplements among pregnant women; and data on intake adherence (Mei, Jefferds, Namaste, Suchdev, & Flores‐Ayala, 2018).

### 3.17 UNICEF supply division support programmes in quality assurance, logistics, and medicines and nutrients

UNICEF supplies products to many countries worldwide and is committed to ensure the quality of the products it supplies. The UNICEF Quality Assurance system is based on standard operating procedures, international standards for quality assurance (including WHO good manufacturing practice system), and the continuous review of product specifications (United Nations Children's Fund, 2016). The standard operating procedures specifically for pharmaceuticals are based on a quality assurance policy guided by the WHO model quality assurance system for procurement, review of dossiers during and supporting documentation (analytical procedures, stability data, sources of APIs); good manufacturing practice inspections as per PIC/S quality system requirements, and quality control by random sampling and analytical testing and systematic pre‐delivery inspections to packaging and documentation (United Nations Children's Fund/WFP/World Health Organization, 2006).

Considerations about classifying MMN and IFA as drugs, dietary supplements, or food could affect availability in countries and inclusion in local list of essential medicines. Usually, classification as drugs requires clinical evidence included in the dossier to the regulatory authority; the registration process could take up to 6 years but minimum 2 years, and claims are limited to indications based on clinical data. For classifications as a dietary supplement, the registration process varies from 1 month to 1 year; there is no requirement for stability data prior to product release on the market, and the claims about product can be broad and may not require clinical trial evidence. To be registered as a food, the focus is on food safety aspects and little emphasis on therapeutic effects of product; usually, there is no registration needed, and the claims about the product can be broad (United Nations Children's Fund, 2016).

### 3.18 SUN movement: Considerations for decision making on different implementation approaches

The SUN (Scaling‐Up Nutrition) movement (http://scalingupnutrition.org/about-sun/the-vision-and-principles-of-sun/) promotes commitments to eliminate malnutrition aligned to stakeholder investments and actions, engaging different actors such as academia, civil society, donors, industry, government, and the UN. SUN promotes health and water sanitation, women empowerment, education, development, agriculture, and social protection.

The aim of the movement is to look for and choose the most efficient combination for sustained equitable impact. The building blocks being technical (efficacy, feasibility, and effective coverage), political (acceptability, success, preference), and financial (cost estimates, cost share, cost‐effectiveness; Scaling‐Up Nutrition Movement, 2015).

### 3.19 Countries' presentations

Each country participating in the meeting had the opportunity to present their scenario related to micronutrient deficiencies and what do they need in order to work together with the support of all the entities participating in this technical meeting.

#### 3.19.1 Tanzania

Recognised the need to create awareness to improve the demand from consumers, to create capital investment, and to improve nutrition technology to limit dependence on imported supplements. They identified the need to improve human resources and strengthen regulatory frameworks.

#### 3.19.2 Nigeria

Prevalence of anaemia in children and women is around 50%. Opportunity for inclusion of MMN is under discussions on national guidelines. They report a strong IFA programme that could facilitate implementing MMN in adults and children, mainly in the north part of the country. They have identified the needs for capacity building and funding.

#### 3.19.3 Zimbabwe

Anaemia and iron deficiency are major public health problems in Zimbabwe. Village health workers provide support to health centre staff for IFA supplementation at the community level. Nutrition has been placed in the global developmental agenda. One of the challenges is to address the food sector and nutrition. Challenges: different formulations in the market, poor adherence due to side effects, religious objectors, and stocks outs.

#### 3.19.4 Zambia

Some of the challenges identified included the difficulties of defining MMN as food or drug at the moment of registration, too many formulations of MMNs on the market, very limited funding to procure MNNS, continuous stock outs, poor understanding of the importance of micronutrients by the general population, lack of definition of micronutrients in local languages, and poor micronutrient intake adherence. The major needs include funding and capacity building for operational research, review of the micronutrient operational strategy, update on guidelines and standards, development of advocacy package for various audiences, and funding for procuring of supplies.

#### 3.19.5 Uganda

The attendance to ANC is high; however, there is low adherence to supplementation programmes. There are also food fortification programmes in place (flour, oil, and salt). There is awareness about MMN; they have created a working group for anaemia, and comprehensive micronutrient deficiency guidelines are being finalised. There is intersectoral action with other interventions that affect health in general, such as mosquito nets, deworming, and supplementation. The needs highlighted for Uganda were to improve communication about the importance and need for supplementation programmes and more research.

#### 3.19.6 Madagascar

In Madagascar, 47% of children under five suffer from malnutrition, and anaemia is the leading cause of maternal mortality. IFA coverage is 8% because 60% of people live far from a health facility. There is a need to strengthen the number and preparation of community workers to reach people who can't access or afford the supplements to be included in programmes.

#### 3.19.7 Panama

Prevalence of anaemia of 23% and ANC coverage of 96%. Ongoing programmes to deliver two tablets as ANC one combined folic acid and iron and other MMN locally produced. Population is concentrated in one city, many indigenous groups with strong cultural differences; this would be the strongest challenge.

#### 3.19.8 Indonesia

Population of 48 million, free ANC, 29% of compliance, with a long history of the IFA fortification programme. The main challenge in Indonesia is acceptability, because there is a gap between provision and consumption. They have tried MMN to see if it is more efficient than IFA in spite of the cost, but they face the same problems.

## 4 CLOSING REMARKS

Discussions sessions during the meeting were focused on the little progress obtained on anaemia control in spite of the big efforts and resources invested. There was a consensus on the need to improve programme implementation prior to consider a transition to MMN supplements. There are various elements of programmes that need to be improved, from financial allocation for supplement forecasting to the development of behaviour communication interventions that can raise awareness among women, health personnel, and decision makers. Building the capacity of staff was considered a key element. There are some successful experiences, but we still need to acknowledge the failures and learn from them to improve course correction.

The WHO has focused efforts to make IFA supplementation an important part of different health packages such as ANC and adolescent health and aims to provide clear guidance to help member states achieve universal health coverage. Basic nutrition interventions should become part of the concept of universal health coverage and quality of care.

Another important topic raised during plenary sessions was about the composition of the product itself and the careful consideration of evidence, or the lack of it, to inform a possible change in the national policies and distribution of the product through the public sector to control the anaemia problem. On one hand, MMN formulations offer a great opportunity at marginal cost to add other nutrients that may improve pregnancy outcomes and that are not considered or measured so far, for example, vitamin D combined or not with calcium. The majority of studies are focused on anaemia, but it is only a part of the maternal nutrition status. There are other outcomes with long term impacts, developmental impacts, beyond anaemia reduction. On the other hand, it was acknowledged that the higher cost of MMN supplements in comparison with IFA may be a challenge in countries with limited budget for nutrition interventions. Innovative transition strategies would be needed to favour the adoption of MMN.

Guidelines on MMN supplementation are necessary but also a policy environment and political will. Other key factors are capacity building, supply management, right formulation, clear regulation of the countries, quality health services, and behaviour change: the demand for nutrition actions.

## 5 CONCLUSIONS

In many countries, little progress has been achieved to ensure universal coverage of IFA supplementation in pregnancy, and it is necessary to address the operational issues that are hindering maternal supplementation programmes as part of ANC. Participants pointed out the concern that the provision of IFA only can limit the potential of ANC programmes particularly when other micronutrient deficiencies appear during pregnancy. Several countries shared their experience in maternal micronutrient supplementation during this meeting showing different level of advances and challenges. In all cases, there is a need to address technical, evidence, and implementation gaps.

Participants agreed on the need for policy development and stronger regulatory frameworks. A favourable policy environment is required for effective procurement, implementation, and assessment of programmes. This could be achieved by the following:

- Using a standard formulation in trials to demonstrate the effects of MMS could increase study comparability and help to better inform programmes. The formulation known as UNIMMAP (UNICEF/WHO/United Nations University (UNU) international MMN preparation) is already tested and may be adopted widely.
- Encouraging countries to consider the feasibility and convenience of including MMN supplements in their Essential Medicines List.
- Addressing trade and procurement barriers that may be impeding policy uptake.
- Strengthening and improving quality assurance and control mechanisms.

It was concluded there was a need for stronger programmatic emphasis on the following areas:

- Equity: Improving the equitable access to nutrition interventions at the different stages of programme design, implementation, and evaluation is key to achieve successful programme outcomes.
- Behaviour communication interventions: Effective behaviour communication interventions are needed in order to strengthen ANC demand and quality and also to engage family or actors to support improved adherence (e.g., addressing men).
- Monitoring and cost/effectiveness evaluation. Micronutrient supplementation during pregnancy can benefit from setting up or strengthening monitoring and surveillance systems and to assess costs at the different stages of the supply chain (e.g., production, distribution, stock, and delivery to users). Assessing achievements and highlighting important learnings are key for effective planning and large scale implementation of interventions at public health level.
- Continuum of care. Women frequently reach pregnancy with micronutrient deficiencies. Implementing cost‐effective interventions before pregnancy (e.g., women health and social empowerment, nutrition sensitive interventions, and nutrition specific interventions aimed at women) is a good strategy to improve maternal health and nutrition.
- Prioritise action plans according to the context. Countries can decide if they keep and strengthen their current IFA supplementation programmes or decide to transition to a MMN supplementation programme.

Finally, participants acknowledged that partnerships and good governance are key factors to making ANC programmes effective, coordinated, and successful. Intersectoral actions must be integrated with maternal child health interventions. Hence, it is essential to engage key stakeholders to gain political commitment and financial support.

## CONFLICTS OF INTEREST

The authors declare that they have no conflicts of interest.

## CONTRIBUTIONS

DE attended the technical meeting and presented a preliminary report of the meeting. MNGC wrote the first draft of the manuscript, attended the technical consultancy meeting, and finalised the manuscript. LMDR participate in the meeting preparation and review the manuscript. All authors approved the final version for publication.
